# Translation and validation of Training Needs Analysis Questionnaire among reproductive, maternal and newborn health workers in Tanzania

**DOI:** 10.1186/s12913-021-06686-9

**Published:** 2021-07-24

**Authors:** Tumbwene Mwansisya, Columba Mbekenga, Kahabi Isangula, Loveluck Mwasha, Eunice Pallangyo, Grace Edwards, James Orwa, Michaela Mantel, Micheal Mugerwa, Leonard Subi, Secilia Mrema, David Siso, Edna Selestine, Marleen Temmerman

**Affiliations:** 1 School of Nursing and Midwifery, the Aga Khan University, Dar es Salaam, Tanzania; 2 School of Nursing and Midwifery, the Aga Khan University, Kampala, Uganda; 3 Department of Population Health, the Aga Khan University, Nairobi, Kenya; 4 Aga Khan Health Services, Mwanza, Tanzania; 5 Community Development, Ministry of Health, Gender, Elderly and Children, Dodoma, Tanzania; 6 Regional Reproductive and Child Health Coordinator, Mwanza, Tanzania; 7 Aga Khan Foundation, Dar es Salaam, Tanzania

**Keywords:** Validation, Training need analysis, Reproductive, Maternal, Newborn, Healthcare workers

## Abstract

**Background:**

Continuous professional development (CPD) has been reported to enhance healthcare workers’ knowledge and skills, improve retention and recruitment, improve the quality of patient care, and reduce patient mortality. Therefore, validated training needs assessment tools are important to facilitate the design of effective CPD programs.

**Methods:**

A cross-sectional survey was conducted using self-administered questionnaires. Participants were healthcare workers in reproductive, maternal, and neonatal health (RMNH) from seven hospitals, 12 health centers, and 17 dispensaries in eight districts of Mwanza Region, Tanzania. The training needs analysis (TNA) tool that was used for data collection was adapted and translated into Kiswahili from English version of the Hennessy-Hicks’ Training Need Analysis Questionnaire (TNAQ).

**Results:**

In total, 153 healthcare workers participated in this study. Most participants were female 83 % (*n* = 127), and 76 % (*n* = 115) were nurses. The average age was 39 years, and the mean duration working in RMNH was 7.9 years. The reliability of the adapted TNAQ was 0.954. Assessment of construct validity indicated that the comparative fit index was equal to 1.

**Conclusions:**

The adapted TNAQ appears to be reliable and valid for identifying professional training needs among healthcare workers in RMNH settings in Mwanza Region, Tanzania. Further studies with larger sample sizes are needed to test the use of the TNAQ in broader healthcare systems and settings.

## Background

Reproductive, maternal, newborn, child, and adolescent healthcare remains a challenge in many low-income countries. For example, Tanzania failed to meet the Millennium Development Goal (MDG) target of reducing the maternal mortality ratio (MMR) to 228 per 100,000 live births by 2015 [1]. The MMR in Tanzania remains unacceptably high, despite the slight decline between the 1990 s (578 per 100,000 live births) and 2016 (556 per 100,000 live births) [1, 2]. In addition, the national neonatal mortality rate (NMR) remains high, at 25 deaths per 1,000 live births [2].

Although there are many drivers of poor maternal and newborn health, low quality of reproductive healthcare services was identified as an important contributor [3]. The poor quality of delivery services may be associated with shortage of skilled birth attendants [4]. Previous studies estimated that 20–21 % of neonatal deaths could be prevented by healthcare workers’ promotion of simple, evidence-based practices (e.g., exclusive breastfeeding and hand washing) and prevention of hypothermia and infection [5, 6]. Other studies indicated that healthcare workers were inadequately supported in terms of training and remuneration, lack of skills mix, and poor retention strategies [7]. Moreover, the Tanzania Government budget allocation to reproductive, maternal, and neonatal health (RMNH) is limited and inconsistent [3]. Taken together, these factors lead to inadequate quality of healthcare systems.

Continuous professional development (CPD) may help address this issue, as it has been reported to enhance healthcare workers’ knowledge and skills, improve retention and recruitment, improve the quality of patient care, and reduce patient mortality [8, 9]. In addition, skilled birth attendants play a vital role in ensuring optimal RMNH practices by communicating with care and respect and having the requisite knowledge and technical proficiency [5]. It is therefore important to assess the training needs of RMNH staff to facilitate the development of capacity building interventions to bridge existing skill-related gaps, thereby improving the quality of healthcare systems.

An important tool in identifying healthcare workers’ knowledge and skills gaps and establishing future CPD training profiles is training needs analysis (TNA) [8]. Context-specific TNA can facilitate accurate assessment of healthcare workers’ training needs for successful RMNH programs [10]. Furthermore, a validated TNA tool to assess training needs in low-income countries is needed to identify the context-specific underdeveloped skills, insufficient knowledge, or inappropriate attitudes among healthcare workers. Despite the contribution of CPD in reducing the MMR and NMR, research shows that TNA in many organizational settings has been unsystematic or used tools that were not validated [11]. This highlights the need for validated TNA tools to facilitate the design of effective CPD programs.

The Aga Khan Development Network is working with the Tanzania Ministry of Health, Community Development, Gender, Elderly and Children to improve access to RMNH in Mwanza Region, Tanzania (IMPACT project). This 4-year project (2017–2021) focused on reproductive health services, and aimed to accelerate the reduction of maternal and newborn mortality in Tanzania by addressing major RMNH challenges in eight districts of Mwanza Region. The IMPACT project combines training with mentorship interventions that have been reported to be important strategies to support skills and capacity transfer among in-service healthcare workers [12], which in turn improves RMNH outcomes [13]. In the project’s baseline survey, we conducted TNA among healthcare workers in RMNH in Mwanza Region. The IMPACT project team developed a TNA tool based on adapting items from the Hennessy-Hicks Training Needs Analysis Questionnaire (TNAQ) [14], which was developed in 1996 to evaluate training needs and priorities of healthcare professionals. The TNAQ has been used in both developing and developed countries [14–16]. This study aimed to validate the adapted TNAQ in the local Tanzanian context, with the goal of facilitating development of effective needs-based CPD programs to improve healthcare workers’ competence in the delivery of RMNH and child and adolescent healthcare services in Mwanza Region.

## Materials and methods

### Study Area

Details of the study settings have been reported elsewhere [17]. Briefly, this study was conducted in Mwanza Region, which is in the northern part of Tanzania bordering Lake Victoria. The 2012 national census indicated Mwanza Region had a population of 2,772,509 people in an area of 35,187 km^2^ [2]. The average annual population growth rate for 2002–2012 was 3.0 %, making Mwanza among eight regions in Tanzania with a high growth rate. The Tanzania Human Development Report ranked Mwanza Region as 13th among Tanzania’s 35 regions, with one-third of the population living in severe poverty (32.8 %) and one-fifth of the population vulnerable to poverty (19.7 %) compared with national averages of 31.3 and 18.2 %, respectively [1].

The region is part of the Lake Zone, where the MMR was 453 deaths per 100,000 live births and the under-five mortality rate was 88 deaths per 1,000 live births in the 10-year period preceding the 2015/16 Tanzania Demographic and Health Survey; these rates failed to meet Tanzania’s MDG targets [2]. Available data on the NMR of Mwanza Region in 2015 showed there were 29 deaths per 1,000 live births, which was higher than the national average of 25/1,000 [2].

National data indicate that only 50.7 % of pregnant women attended at least the four recommended health facility visits for focused antenatal care during their last pregnancy [2]. Health facility deliveries in Mwanza Region account for 63.6 % of deliveries on average, although there are persisting large disparities, with 87 % of deliveries occurring in facilities in urban areas versus 54.7 % in rural areas [2]. Given the poor RMNH indicators, Mwanza Region is one of five regions in Tanzania that have been prioritized by the Government [2]. Understanding the training needs of healthcare workers in Mwanza Region formed an important entry point for the IMPACT project in seeking to improve RMNH indicators. This provided the rationale for validating the TNAQ in this region.

### Design

We conducted a cross-sectional survey using self-administered questionnaires. The survey involved healthcare workers in RMNH at selected health facilities in all eight districts of Mwanza Region, Tanzania.

### Study population

All adult healthcare workers responsible for RMNH service provision that were present in the selected facilities at the time of this survey were eligible to participate.

### Eligibility and sampling

Eligible participants were healthcare workers responsible for RMNH services. The selected healthcare workers were from 36 stratified health facilities randomly sampled from the 80 IMPACT project-supported sites. These healthcare facilities comprised seven district hospitals, 12 health centers, and 17 dispensaries. All healthcare workers providing RMNH services who were available at the time of the study visit were included. In total, 153 healthcare workers were used to validate the TNAQ, giving an item to participant ratio of 1:3 [18]. This ratio related to the finite sample of healthcare workers in RMNH in the selected facilities. Although some studies suggested an item to participant ratio of 1:10 was appropriate, other studies used ratios of 1:2 [18]. Therefore, the sample size used in this study was considered adequate.

### Data collection tool

The TNAQ was designed for RMNH providers at the primary (dispensary and health center) and secondary (health center and district and designated district hospital) levels. As noted above, the tool was adapted from that developed by Hennessy-Hicks [14, 19], which has been psychometrically tested for reliability and validity and adopted by the World Health Organization (WHO) [19]. Several other TNA questionnaires were considered, including the Professional Nurse Self-Assessment Scale of Clinical Core Competencies [20], Addiction Medicine Training Needs Analysis Scale [21], and Community Competence-based Questionnaire [22]. However, the Hennessy-Hicks TNAQ was selected because it was developed specifically to evaluate healthcare professionals’ training requirements and facilitate subsequent use of the findings to prioritize and meet local training needs [19]. The Hennessy-Hicks questionnaire measures a range of skills including clinical, managerial, administrative, and research audit activities. Responses to the TNAQ are on a seven-point Likert scale: “not at all important” (1), “slightly important” (2), “quite important” (3), “moderately important” (4), “important” (5), “very important” (6), and “extremely important” (7). Currently, there is no validated and standardized tool for assessing training needs in RMNH in Tanzania. There is also a shortage of skilled birth attendants in the region, meaning that the few who are available must perform a range of activities. Therefore, validation of the Hennessy-Hicks TNAQ was deemed necessary to capture the multiple responsibilities of RMNH personnel.

### Translation

 Two bilingual health professionals experienced in reproductive health and research provided a forward translation of the TNAQ to the Kiswahili language. This was followed by back translation by a bilingual non-health professional. The two versions were compared, and suggestions for modification incorporated in the Kiswahili version. The adapted TNAQ was then pilot tested with 12 healthcare professionals working in reproductive health and management. The feedback received from the pilot study was incorporated in the final adapted TNAQ. Minimum translation criteria were applied [23].

### Data collection procedure

Data were collected in August 2017. During data collection, the person in-charge of each selected facility identified RMNH personnel for participation. The paper-based TNAQ was self-administered, and responses were confidential. The Questionnaires were administered to participants during their working hours to maximize their participation. Participants were requested to assess their own performance and rate the perceived importance of specific RMNH services/activities. Specifically, the questionnaire asked participants how important each RNMH-related activity was to the successful performance of their work and how good they considered their performance in each activity. Participants were also asked to identify areas in which they most wanted to receive additional training, and to note the training that they had most recently completed. Research assistants were available to answer questions and clarify elements of the questionnaire as needed. Returned questionnaires were checked for completeness and accuracy before the research team left each health facility.

### Data management

SPSS version 20.0 was used for data entry and statistical analyses. Data from the questionnaires were reviewed to identify consistencies and differences, coded, and quantified. The data were then manually entered into a password-protected database via an entry screen that performed validation checks for accuracy. Missing data were excluded during analysis. Analysis of Moment Structures (AMOS) software embedded in SPSS version 20 was used to confirm the factor structure of the TNAQ using an exploratory analysis.

### Data analysis and interpretation

The adapted TNAQ was evaluated for both reliability and validity. Reliability was used to determine the stability and equivalence of sets of TNAQ items. Although reliability is necessary, it is not sufficient to determine the validity of an instrument [21]. Therefore, we also assessed the validity of the TNAQ to determine the degree to which the instrument could measure what it was is supposed to measure.

### Reliability of the scale

The internal consistency reliability was determined by Cronbach’s alpha. A Cronbach’s alpha of 0.7 is considered to indicate acceptable reliability [21]. Moreover, the individual quality effect of each item was assessed using Cronbach’s alpha to test the reliability if an item was deleted. Split-half reliability was calculated by randomly splitting the items into two sets to determine the consistency of the TNAQ across sub-groups. A Guttman split-half coefficient of ≥ 0.80 indicates good internal consistency [21]. Finally, inter-rater reliability was used to evaluate the performance of the measure across different raters, and was determined by the intra-class correlation coefficient [24]; a coefficient ≥ 0.7 indicates an acceptable level of inter-rater reliability.

### Validity of the scale

#### Face and content validity

The Hennessy-Hicks instrument has been adapted to assess the training needs of different health care practitioners in a range of cultural contexts [14, 19, 25]. However, it has not previously been validated in the Tanzanian context where the culture may differ from countries where the instrument has been used. In adapting the TNAQ for this study, pooled items were obtained from a literature review and the opinion of experts experienced in the reproductive health field. The pooled items were validated by three experts with expertise in teaching, reproductive health, research, and the local culture including customs, traditions, and the local language (Kiswahili). The TNA tool divides the items into broad categories, allowing for both intra-category and inter-category comparison of training needs.

#### Criterion validity

We assessed concurrent validity, which is one of three types of criterion validity (predictive validity, concurrent validity, and postdictive validity) [26]. The TNAQ was found to have acceptable concurrent validity.

#### Construct validity

The sampling adequacy was determined by the Kaiser-Meyer-Olkin (KMO) measure and the suitability of factorization was assessed using Bartlett’s test of sphericity [27]. Construct validity was evaluated using exploratory factor analysis (EFA) and confirmatory factor analysis (CFA). The factor structure was first evaluated with EFA, which was conducted using principal axis factoring estimation with promax rotation. This determines the underlying factor structure of the items, and has the advantages of being fast and conceptually simple [28]. The criteria applied for factor retention were: (a) eigenvalues greater than 1.0, (b) the percentage of total variance explained, (c) scree plot, and (d) factors with loadings above 0.40. Items with a loading below 0.4 and those with cross loading over 0.4 were deleted. CFA was used to validate the factor structure obtained from the EFA. The CFA was determined by the factor loading > 0.4 by using the AMOS statistical program. The CFA indicated the adequacy of the data in the model and the appropriate fit of the structural model for the healthcare workers who are the study population. The variance and covariance matrix of the 49 items was determined by maximum likelihood minimization function and the 3-factors model was assumed basing on our analysis. To adjust the scaling of the factors to that of the indicators, a fixed 1.0 factor loading and a fixed measurement error variance was applied. Model fit was considered acceptable if χ^2^/df < 2, comparative fit index (CFI) > 0.9, root mean square error of approximation (RMSEA) < 0.04 [27, 29].

#### Convergent and discriminant validity

##### Convergent Validity

Convergent validity refers to how variables on a single factor are correlated [30]. We assessed the convergent validity of the adapted TNAQ with the average variance extracted (AVE) and composite reliability (CR). The AVE and CR were calculated manually using the formula suggested by Hair et al., [31]. An AVE > 0.5 and CR > 0.7 indicate convergent validity. Moreover, convergent validity was confirmed by the factor loadings. The significance of factor loading is based on the sample size. Generally, the smaller the sample size, the higher the factor loadings [18]. For example, a sample of 50 participants may require a sufficient factor loading of 0.75 whereas a sample of 350 may require a sufficient loading of 0.3. Therefore, the sample size of 153 participants used in this study required factor loadings of 0.4 to be sufficient.

##### Discriminant validity

To establish discriminant validity, the maximum shared variance (MSV) and average shared variance (ASV) were calculated, and their values compared. A MSV value less than the ASV value indicates discriminant validity [30]. Furthermore, the correlation matrix was examined with correlations between factors < 0.7 indicating discriminant validity.

## Results

Participants were 153 healthcare workers. Most participants were female (8.73 %, *n* = 128) and the average age was 39 years. Nurses formed the largest group of participants (76 %, *n* = 115), and the mean time in RMNH was 7.9 years. Moreover, most participants indicated they had experience of 0–10 years in both general healthcare services and RMNH. Participants’ sociodemographic details are presented in Table 1.

**Table 1:** Demographic characteristics and clinical experience of study participants working in reproductive health in Mwanza (*n* = 153)

Characteristic	Dispensary (*n* = 29)	Health center (*n* = 53)	Hospital (*n* = 70)	Total (*n* = 152)^a^
Mean age (years)	37.4	38.0	39.9	38.8
Sex (M, F)	8, 21	8, 45	9, 61	25, 128
Mean years in service	12.9	13.4	14.0	13.59
Mean years in RMNH	8.1	10.1	6.1	7.9
**Cadre**				
Assistant medical officers	1 (3 %)	1 (2 %)	1 (1 %)	3 (2 %)
Clinical officers	0 (0 %)	2 (4 %)	6 (9 %)	8 (5 %)
Enrolled nurses	8 (28 %)	22 (42 %)	23 (31 %)	53 (35 %)
Registered nurses	12(41 %)	22 (42 %)	10 (14 %)	62 (41 %)
Medical attendant	8 (28 %)	6 (11 %)	12 (17 %)	26 (17 %)
**Clinical experience** **(years)**	**In service** **n (%)**	**In RMNH** **n (%)**		
0–10	90 (58.8)	80 (80.4)		
11–20	18 (11.8)	11 (7.2)		
21–30	24 (15.7)	13 (8.5)		
30+	19 (12.4)	7(3.9)		

^a^One participant had missing demographic data and was excluded from this analysis

### Reliability of the adapted TNAQ

The Cronbach’s alpha of the TNAQ was 0.954, showing internal consistency. Moreover, deletion of each item individually resulted in a Cronbach’s alpha ≥ 0.953. The split-half reliability was also good, with a Guttman split-half coefficient of 0.84. The intra-class correlation coefficient was 0.954 showing inter-rater reliability. Table 2 presents details of the internal consistency for individual items.

**Table 2:** Internal consistency of the items in the adapted Training Needs Analysis Questionnaire

Item number	Scale mean if item deleted	Scale variance if item deleted	Corrected item-total correlation	Cronbach’s alpha if item deleted
1.	284.75	998.294	0.513	0.954
2.	284.81	1005.931	0.443	0.954
3.	284.74	993.973	0.551	0.954
4.	284.85	980.934	0.555	0.953
5.	284.53	998.081	0.594	0.954
6.	284.64	1000.593	0.460	0.954
7.	284.47	994.166	0.514	0.954
8.	284.40	1002.037	0.537	0.954
9.	284.86	990.278	0.445	0.954
10.	284.67	986.753	0.501	0.954
11.	284.44	991.086	0.588	0.953
12.	284.56	987.599	0.504	0.954
13.	284.95	974.630	0.464	0.954
14.	284.45	983.395	0.653	0.953
15.	284.42	990.877	0.581	0.953
16.	284.45	983.771	0.642	0.953
17.	284.49	985.397	0.584	0.953
18.	284.27	995.447	0.713	0.953
19.	284.39	987.983	0.592	0.953
20.	284.31	1000.097	0.585	0.954
21.	284.64	989.293	0.652	0.953
22.	284.58	984.262	0.567	0.953
23.	284.69	984.713	0.634	0.953
24.	284.33	994.274	0.729	0.953
25.	284.61	984.035	0.734	0.953
26.	284.79	982.476	0.640	0.953
27.	284.81	986.261	0.565	0.953
28.	284.75	976.119	0.621	0.953
29.	285.86	970.877	0.376	0.956
30.	285.93	970.064	0.365	0.957
31.	284.86	968.894	0.684	0.953
32.	284.85	966.985	0.713	0.953
33.	284.83	973.492	0.658	0.953
34.	284.90	982.280	0.523	0.954
35.	284.64	975.753	0.709	0.953
36.	284.83	972.945	0.644	0.953
37.	284.62	982.768	0.599	0.953
38.	284.42	998.520	0.679	0.953
39.	284.35	997.152	0.628	0.953
40.	285.08	991.114	0.405	0.954
41.	285.03	986.623	0.479	0.954
42.	284.55	986.660	0.717	0.953
43.	284.64	996.952	0.466	0.954
44.	284.69	992.197	0.506	0.954
45.	284.52	996.748	0.485	0.954
46.	284.47	989.722	0.607	0.953
47.	284.84	992.598	0.458	0.954
48.	284.91	986.496	0.455	0.954
49.	284.67	986.223	0.648	0.953

### Content validity

Pooled items that were obtained from the literature review and expert opinion were validated by an expert panel with expertise in teaching, reproductive health, research, and local culture including customs, traditions, and the local language (Kiswahili) that is spoken by 95 % of Tanzanians. All items were found to be adequate and clear during translation (expert opinion) and pilot testing. This indicated good content validity. In terms of face validity, the adapted TNAQ had variables in a single factor that were related and easy to label. We found three factors: general reproductive health, intraoperative care, and comprehensive emergency obstetric and newborn care (CEmONC). These labels were related to the similar focus of the items in a single factor. Assessment of the criterion validity showed the adapted TNAQ had acceptable validity and reliability, similar to the original Hennessy-Hicks instrument. In terms of convergent validity, the AVE was 0.5 and the CR was 0.6. Moreover, all items had factor loadings above 0.4. The three factors each included variables that were highly inter-correlated (Table 3). Finally, examination of discriminant validity showed that the MSV was 0.49, which was below the AVE of 0.5. Discriminant validity was also determined by factor correlation matrix. The Pearson correlation coefficients between factors were < 0.7, indicating the adapted TNAQ had discriminant validity. Detailed results are provided in Table 3.

**Table 3:** Factor correlation matrix for the adapted Training Needs Analysis Questionnaire

Factor	General reproductive health	Intraoperative care	CEmONC
General reproductive health	1	0.310	0.431
Intraoperative care	0.310	1	0.431
CEmONC	0.431	0.407	1
TNA total scores	0.988	0.444	0.506

*CEmONC* comprehensive emergency obstetric and newborn care

^a^Correlations significant at the 0.01 level (two-tailed)

### Construct validity

**Fig. 1:**
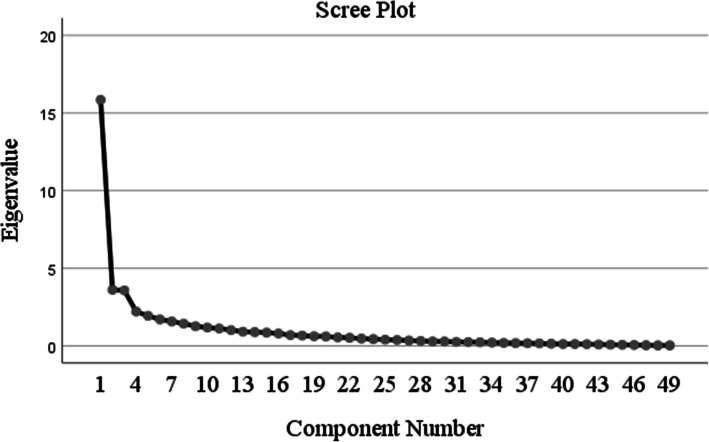
Scree plot with eigenvalues to indicate the distribution of principal components

The EFA produced three factors with eigenvalues of > 1.0 and sampling adequacy of 85 %. Those factors were responsible for 36.77 % of the variance. The rotation converged in 11 iterations. An examination of the KMO measure of sampling adequacy suggested that the sample was factorable (KMO = 0.85). The Barlett’s test of sphericity was significant (χ^2^ = 4860.715, df 1176, *p* < 0.0001). Details for the factor loadings of TNAQ items are shown in Fig. 1.

As shown in Table 4, the TNAQ retained all 49 items and displayed a three-factor structure. Factor 2 included item number 13 (CEmONC activities) and was labeled CEmONC. Factor 3 included items 29 (surgical care) and 30 (anesthetic care) and was labeled intra-operative care. All other remaining items formed Factor 1, which was labeled general RMNH activities.

**Table 4:** Principal component categorization indicating three factors from study participants (*n* = 153)

SN0	Items	1	2	3
1	Understanding gender equality issues relating to reproductive, maternal, child, and adolescent health	0.557		
2	Delivering gender sensitive reproductive, maternal, child, and adolescent health services (e.g., providing privacy for consultations, gender sensitive counselling approaches, involvement of men)	0.505		
3	Providing client/patient friendly reproductive, maternal, child, and adolescent health services	0.607		
4	Understanding and using maternal, newborn, and child health (MNCH) score cards [1]	0.621		
5	Providing focused antenatal care (FANC) according to WHO guidelines	0.652		
6	Offering malaria diagnosis with rapid diagnostic testing (RDT)	0.512		
7	Providing malaria treatment in pregnancy	0.557		
8	Providing education and counselling around voluntary counselling and testing (VCT) for HIV/AIDS	0.605		
9	Providing education, counselling, and support around HIV/AIDS prevention, care, and management for adolescents	0.491		
10	Competently managing uncomplicated deliveries	0.552		
11	Competently using the partograph for every woman in labor	0.626		
12	Competently providing basic emergency obstetric and newborn care (BEmONC) including: o Parenteral antibiotics o Parenteral uterotonic drugs o Parenteral anticonvulsants o Manual removal of retained placenta o Removal of retained products of conception o Instrumental vaginal delivery Basic neonatal resuscitation	0.508		
13	Competently providing comprehensive emergency obstetric and newborn care (CEmONC) o All BEmONC signal functions plus o Surgery o Blood transfusion		0.467	
14	Competently managing severe intra- and postpartum hemorrhage	0.687		
15	Responding effectively to women suffering from severe pre-eclampsia and eclampsia	0.612		
16	Effectively resuscitating newborns using the newborn bag and mask (HBB-Helping Babies Breathe)	0.681		
17	Identifying dangerous signs and complications in childbirth and effectively managing maternal and newborn referral for further investigations or treatment	0.631		
18	Providing education and counselling on prevention of mother to child transmission of HIV (PMTCT)	0.765		
19	Effectively managing PMTCT treatment of HIV positive pregnant women, mothers, and infants	0.637		
20	Providing education, counselling, and support to mothers in early initiation of breast feeding (within 1 h of delivery) and exclusive breast feeding for 6 months	0.650		
21	Implementing the maternal, infant, and young child nutrition (MIYCN) program	0.681		
22	Offering the Tanzania expanded program for immunization (EPI)	0.600		
23	Understanding vaccine management and logistics (cold chain maintenance)	0.668		
24	Being proficient on injection safety and infectious waste management	0.777		
25	Providing family planning services to women and men in a union	0.770		
26	Providing family planning services to unmarried/single women and men	0.675		
27	Providing information, education, counselling, or family planning services to adolescents	0.602		
28	Providing care and education for cervical cancer screening and treatment	0.617		
29	Feeling confident in providing surgical care (including cesarean section)			0.533
30	Feeling confident in providing anesthetic care			0.572
31	Identifying cases of sexual and gender-based violence and knowing how to make appropriate referrals	0.671		.
32	Providing counselling, care, and support for women who are subject to gender based violence	0.721		
33	Planning and organizing an individual patient’s care	0.653		
34	Evaluating patients’ psychological and social needs	0.504		
35	Implementing effective infection control strategies	0.712		
36	Implementing effective disease surveillance and reporting	0.639		
37	Organizing your own time effectively	0.623		
38	Personally coping with change in the health service delivery	0.721		
39	Working as a member of a team	0.660		
40	Assuming a leadership role	0.435		
41	Developing leadership skills	0.503		
42	Mentoring and guiding other staff	0.758		
43	Supervision and management of community health workers	0.487		
44	Training of community health workers	0.514		
45	Undertaking effective data reporting and monitoring of service delivery	0.500		
46	Statistically analyzing your own data and using health facility data to understand local health challenges and inform service delivery	0.635		
47	Identifying research needs and designing locally relevant research	0.470		
48	Accessing research resources (e.g., time, money, information, equipment)	0.450		
49	Actively influencing evidence-based service provision	0.672		

### CFA for TNAQ

CFA was performed using generalized least squares estimation to compare the current and original 3-factor model of the scale. The model was found to be good (χ^2^/df < 3) and the CFI was optimal (CFI = 1). However, the RMSEA was 0.185 indicating a poor model fit; this index was ignored because the other two indices showed a good fit and are commonly used and considered more predictive. The indices and related thresholds for interpretation are summarized in Table 5.

**Table 5:** Measures for confirmability factor analysis indicating model suitability

Indices	Measure observed	Threshold
CFI	1	> 0.95 = best, > 0.90 = traditional, > 0.80 = permissible
χ^2^ (CMIN/DF)	0.000	< 3 = good, < 5 = permissible
RMSEA	0.185	< 0.05 = good, 0.05–0.10 = moderate, > 0.10 = poor

## Discussion

This study aimed to translate and validate the Kiswahili version of TNAQ in RMNH services in Mwanza Region, Tanzania. Identification of training needs is an important step in developing effective and impactful capacity building interventions to bridge existing skill gaps among healthcare workers [23]. Rather than generating a new tool, which is time and resource intensive, we adapted and validated the Hennessy-Hicks TNAQ [19], which has been widely used in identifying training needs for a range of healthcare workers and is recognized by the WHO [19]. Adapting a TNA tool rather than constructing a new one is an approach used in other health-related specialties [32].

Our findings indicated that the adapted TNAQ had excellent reliability (internal consistency: Cronbach’s alpha 0.954) [33], split-half reliability (Guttman split-half coefficient 0.84) [34], and inter-rater reliability (intra-class correlation coefficient 0.954) [21]. The convergent and discriminant validity of the scale were determined by the AVE, CR, and MSV. The AVE was 0.5, which was an acceptable value (≤ 0.5) for convergent reliability, and the CR was 0.6, which indicated acceptable convergent reliability for an exploratory survey instrument. The MSV of 0.49 was less than the AVE value (0.5), and the correlations between factors was less than 0.7, confirming the model had discriminant validity.

The internal consistency, split-half and intra-rater reliability of the TNAQ was determined. The TNAQ was found with the internal consistency as measured by Cronbach’s Alpha of 0.954 Indicating excellent reliability [33]. The Split-half reliability as measured by Guttman Split-half Coefficient was found to be 0.84 that demonstrates a very good level of internal consistency across sub-sets of the TNAQ. The Guttman Split-half Coefficient of 0.8 indicates a very good level and 0.6 to 0.7 represents the acceptable level however the values of higher than 0.95 may indicate redundancy of the tool therefore are not necessary [34]. The inter-rater reliability as determined by Intra-classes Correlation Coefficient of 0.954 was found, indicating excellent intra-rater reliability as it higher than the acceptable level of 0.7 [21]. Although the content validation procedure for the instrument in this study differed from a previous study that translated and validated the Hennessy-Hicks tool conducted in Greece [25], the overall internal consistency of 0.98 it that study was similar to our study. In addition, although the present study did not test for reproducibility, all reliability indices were within the acceptable threshold, indicating the adapted TNAQ was a consistent tool for assessing RMNH training needs in the Tanzanian context. It is important for a tool to have reliability to be acceptable, but it also needs to be valid [35, 36].

The TNAQ had acceptable face and content validity. We found a three-factor structure that reflected RMNH training needs. This differed from the five categories in the original TNAQ: (research/audit, communication/teamwork, administrative/technical, management/supervisory, and clinical) [14]. This difference may be attributable to the focus of the tools; our adapted TNAQ focused on RMNH whereas the original TNAQ focused on general healthcare workers’ training needs. The language and content of the translated questionnaire were clear and reflected Tanzanian culture. Most Tanzanians share key cultural aspects, such the Kiswahili language (spoken by about 95 % of Tanzanians). Moreover, expression of culture and training needs may reflect those in other low-income countries with similar needs and cultural backgrounds [12]. Cross-cultural adaptation of an instrument is important in standardizing data that can be used in clinical, teaching, and policy analysis in the international arena. Numerous studies from different contexts and cultural backgrounds have reported similar psychometric properties for versions of the TNAQ [21, 25, 37], indicating the TNAQ has acceptable criterion validity. Therefore, the findings of this study suggested that the TNAQ that considered the Tanzanian national guidelines on RMNH care and participants’ culture (including Kiswahili) had an acceptable design and validity.

The convergent and discriminant validity were assessed by both calculated correlations and CFA. The EFA-derived structure was validated by CFA. The correlation coefficients and CFA indices were generally acceptable, indicating the model was suitable. A model is considered good if the X^2^/df = 0.000 is less that the recommended values of less than 3. The chi-square statistic in our structured equation model with a statistically significant p-value indicated the model did not fit the data adequately. Chi-square statistics are sensitive to sample size; therefore, given the small sample size used in this study, it indicates that there was insufficient power to detect differences between competing models [38]. However, use of the chi-square statistic has been debated, with some scholars suggesting chi-square tests are not reliable because of sample size sensitivity, and instead encourage the use of multiple fit indices for a holistic view of the data [38–41] as they are less sensitive to the sample size [40]. Other studies suggested that the inferences based on p-values between large and small sample sizes may be the same but the chi-square values may differ significantly [38].

Our CFI value of 1 was higher than the recommended level (> 0.95), but the RMSEA of 0.185 indicated that it was not a best fit (a smaller value indicates better model fit). The CFI does not give any useful information when RMSEA is more than 0.15 [42]. However, most indices were within the acceptable threshold indicating the instrument had excellent construct validity. Moreover, although there are several types of validity, construct validity is regarded the most important as it forms the basis of other types of validity and is scientifically viewed as the whole of validity [43]. We found the construct validity was acceptable, indicating that the adapted TNAQ was a valid instrument. This finding was similar to another study that found the tool had acceptable validity [25]. It was also similar to the original Hennessy-Hicks instrument [14], which has been psychometrically tested for reliability and validity and adopted by the WHO [14, 19]. Therefore, the adapted TNAQ was scientifically validated to have potential to provide baseline data for identifying training needs among RMNH personnel in primary healthcare settings in Mwanza Region, Tanzania.

There were some limitations to this study that should be considered. First, this study was cross-sectional and only provided a snapshot in-time. The reliability and validity of competence (including behavior and attitudes) in the healthcare industry can better be measured on a continuum-over-time basis [44, 45]. Therefore, further studies that explore test-retest reliability and validity over time are warranted to validate the present findings. Second, there was a disproportionate number of items across the identified factors. Further studies could try to reduce the size of the instrument to keep it practical while maintaining the factor structure and its psychometric properties. Third, the sample size was relatively small and sufficient factor loading depends on the sample size. This limited the division of the dataset into subgroups; the item to participant ratio could therefore have been insufficient for factor loading. Moreover, the interpretation of the findings should take into consideration the inadequate sample size for providing robust estimates, especially for the latent variables included in the model and the participants’ responses were clustered around the higher rank of Likert scale. Finally, some participants did not respond to all items. Therefore, efforts should be made to recruit targeted participants that match the measured level of competence in further studies. However, this did not affect the validity and reliability of this study as missing data were excluded from the analysis.

## Conclusions

The psychometric properties indicated that the Kiswahili translated and adapted TNAQ to be reliable and valid for identifying CPD needs for healthcare workers in the RMNH context at all levels of healthcare settings in Mwanza Region, Tanzania. The sufficient discriminative and evaluative psychometric properties provides evidence for further use of adapted, Kiswahili translated and validated TNAQ in RMNH in Tanzanian context. However, the applicability of the adapted TNAQ in the wider healthcare system remains unclear. Further studies with large sample sizes are required to test the use of the TNAQ in the wider healthcare system and learning opportunities.

## Data Availability

The data that support the findings of this study are available from the Aga Khan University Monitoring and Evaluation Research Unit (MERL). There are some restrictions to the availability of these data due to license, so the data are not publicly available. However, the data may be made available from the authors upon reasonable request and with permission of the Aga Khan University MERL.

## Abbreviations

BEmONC: Basic emergency obstetric and newborn care
CEmONC: Comprehensive emergency obstetric and newborn care
CPD: Continuing professional development
TNA: Training needs analysis
IMPACT project: Improving access to reproductive, maternal and newborn health in Tanzania
TNAQ: Training Needs Analysis Questionnaire
RMNH: Reproductive, maternal, and newborn health
MMR: Maternal mortality rate
NMR: Neonatal mortality rate
WHO: World health organization
CFI: Comparative fit index
RMSEA: Root mean square error of approximation
CR: Composite reliability
AVE: Average variance extracted
EFA: Exploratory factor analysis
CFA: Comparative factor analysis
